# Cerebellar fastigial nucleus: from anatomic construction to physiological functions

**DOI:** 10.1186/s40673-016-0047-1

**Published:** 2016-05-03

**Authors:** Xiao-Yang Zhang, Jian-Jun Wang, Jing-Ning Zhu

**Affiliations:** State Key Laboratory of Pharmaceutical Biotechnology and Department of Biological Science and Technology, School of Life Sciences, Nanjing University, 163 Xianlin Avenue, Nanjing, 210023 China

**Keywords:** Fastigial nucleus, Somatic motor control, Ocular motor control, Feeding control, Cardiovascular control, Respiratory control, Spinocerebellar ataxias, Somatic-nonsomatic integration

## Abstract

Fastigial nucleus (FN) is the phylogenetically oldest nucleus in the cerebellum, a classical subcortical motor coordinator. As one of the ultimate integration stations and outputs of the spinocerebellum, the FN holds a key position in the axial, proximal and ocular motor control by projecting to the medial descending systems and eye movement related nuclei. Furthermore, through topographic connections with extensive nonmotor systems, including visceral related nuclei in the brainstem, hypothalamus, as well as the limbic system, FN has also been implicated in regulation of various nonsomatic functions, such as feeding, cardiovascular and respiratory, defecation and micturition, immune, as well as emotional activities. In clinic, FN lesion or dysfunction results in motor deficits including spinocerebellar ataxias, and nonmotor symptoms. In this review, we summarize the cytoarchitecture, anatomic afferent and efferent connections, as well as the motor and nonmotor functions of the FN and the related diseases and disorders. We suggest that by bridging the motor and nonmotor systems, the cerebellar FN may help to integrate somatic motor and nonsomatic functions and consequently contribute to generate a coordinated response to internal and external environments.

## Background

The fastigial nucleus (FN), together with the interposed nucleus (in humans, emboliform and globose nuclei) and the dentate nucleus, constitutes the cerebellar nuclei, the final integrated nodes and outputs of the cerebellum except the flocculonodular lobe. Compared with the other cerebellar nuclei, the FN is located nearest to the middle line at the anterior end of the superior vermis, and situated immediately over the roof of the IVth ventricle [1–3]. The size of the FN is smaller than the dentate and interposed nuclei. Magnetic resonance imaging (MRI), susceptibility-weighted imaging, and other anatomical studies have reported that the size of FN is approximately 3-6 mm in width, 3-10 mm in length, and 2-5 mm in height in humans [2–6].

Among the cerebellar nuclei, FN is the phylogenetically oldest nucleus. In evolution, cerebellar nuclei system first appeared in the elasmobranch [7]. Studies on the organization of dogfish cerebellar nuclei revealed that the medial and lateral regions separated by dense fascicles of coarse myelinated fibers constituted the ancient cerebellar nuclei system [8]. These medial and lateral divisions of cerebellar nuclei are retained in amphibian, reptiles, as well as birds [9]. In mammals, three cerebellar nuclei, the fastigial (medial), interposed (interpositus) and dentate (lateral) nuclei, embedding separately in a dense mass of white matter appear. A comparative study on the afferent and efferent organizations of cerebellar nuclei system in different species demonstrated that the cerebellar FN and interposed nucleus of mammals are homologous with the medial and lateral nucleus in the cerebellum in lower tetrapods, respectively [9]. However, the FN is highly conserved throughout mammalian evolution, whereas the interposed nucleus has differentiated into the emboliform and globose nuclei in the human cerebellum [1].

The perfect evolutionary conservation of the FN indicates that it may exert a critical functional role in the cerebellum. It has been well known that FN participates in the axial, proximal and ocular motor control. In fact, accumulating evidence reveals that FN also makes topographic connections with various nonmotor structures and actively participates in regulation of various visceral activities, such as feeding, circulation and respiration, defecation and micturition, and even immunity and emotion [10–12]. Therefore, FN dysfunction or lesion may result in not only motor deficits (such as spinocerebellar ataxias, SCAs), but also nonmotor symptoms or syndromes. In this review, the anatomic construction, motor and nonmotor functions, as well as related diseases of the cerebellar FN are summarized and discussed.

## Review

### The cytoarchitecture of the FN

Diverse classes of neurons are heterogeneously distributed throughout the FN. These neurons vary in their morphological features, projection patterns, transmitter phenotypes as well as intrinsic firing properties. Diameters of neuronal somata in the FN range from 5 to 35 μm [13]. According to their projection patterns, FN neurons can be neatly classified into projection neurons and interneurons, with long axons projecting out of the cerebellar nuclei and short axons connecting only with neurons within the FN, respectively [1, 14]. Based on the transmitter phenotypes, glutamatergic, GABAergic as well as glycinergic neurons have been identified in the FN [1, 14, 15]. In addition, electrophysiological features (e.g. action potential waveform, maximal firing rate and input resistance) can also be used as classification criteria to subdivide FN neurons into two populations. One population shows a complex waveform of afterpotentials, marked by a fast afterhyperpolarization (AHP), an afterdepolarization, and then a slow AHP, and exhibits bursts of high-rate firing which are separated from each other by intervals of quiescence under continuous intracellular injection of hyperpolarizing current [13, 16, 17]. On the contrary, the other population shows a simple afterpotential, marked only by a slow AHP, and turns to be silent rather than burst firing under constant hyperpolarization [13, 16, 17].

Although the exact relationship among morphological features, projection patterns, transmitter phenotypes, and intrinsic firing properties of different neuronal types still needs to be intensively investigated, several overlaps among these classifications by different criteria indicate that FN neurons can be subdivided into at least five distinct populations, i.e., large-sized glutamatergic projection neurons, large-sized glycinergic projection neurons, medium-sized GABAergic projection neurons, and small-sized GABA/glycine interneurons and non-GABAergic interneurons. Large-sized glutamatergic projection neurons (diameters ranging from 20 to 35 μm), which send their axons to various brain regions, are mainly distributed throughout the FN [14]. These glutamatergic projection neurons exhibit double AHP and burst firing pattern under constant hyperpolarization [13, 16, 17]. Large-sized glycinergic projection neurons (diameters ranging from 20 to 35 μm) are located exclusively in the rostral of the FN and send fibers to ipsilateral vestibular nuclei and caudal brainstem [18]. The electrophysiological features of the glycinergic projection neurons resemble those of large glutamatergic projection neurons. Medium-sized GABAergic projection neurons (diameters ranging from 10 to 15 μm) provide feedback signals to the inferior olive. Up to now, the intrinsic electrophysiology of the GABAergic projection neurons remains poorly explored. Small-sized GABA/glycine interneurons (diameters < 10 μm) exhibit single AHP and turn into silent under constant hyperpolarization [13, 16, 17]. This type of neurons confines their axonal terminations strictly in the nucleus and thus is responsible for the signaling connection and integration within the FN [15]. Finally, small non-GABAergic interneurons, presumed glutamatergic, have been recently reported existing in the FN [19]. The non-GABAergic interneurons differ from their GABAergic counterparts in that their action potential repolarization and spontaneous firing are faster (albeit slower than that in the glutamatergic projection neurons). However, the exact neurochemical property and functional role of the non-GABAergic interneurons remain enigmatic [14, 19].

### The afferent and efferent connections of the FN

#### Organization of FN afferent pathways

**Fig. 1:**
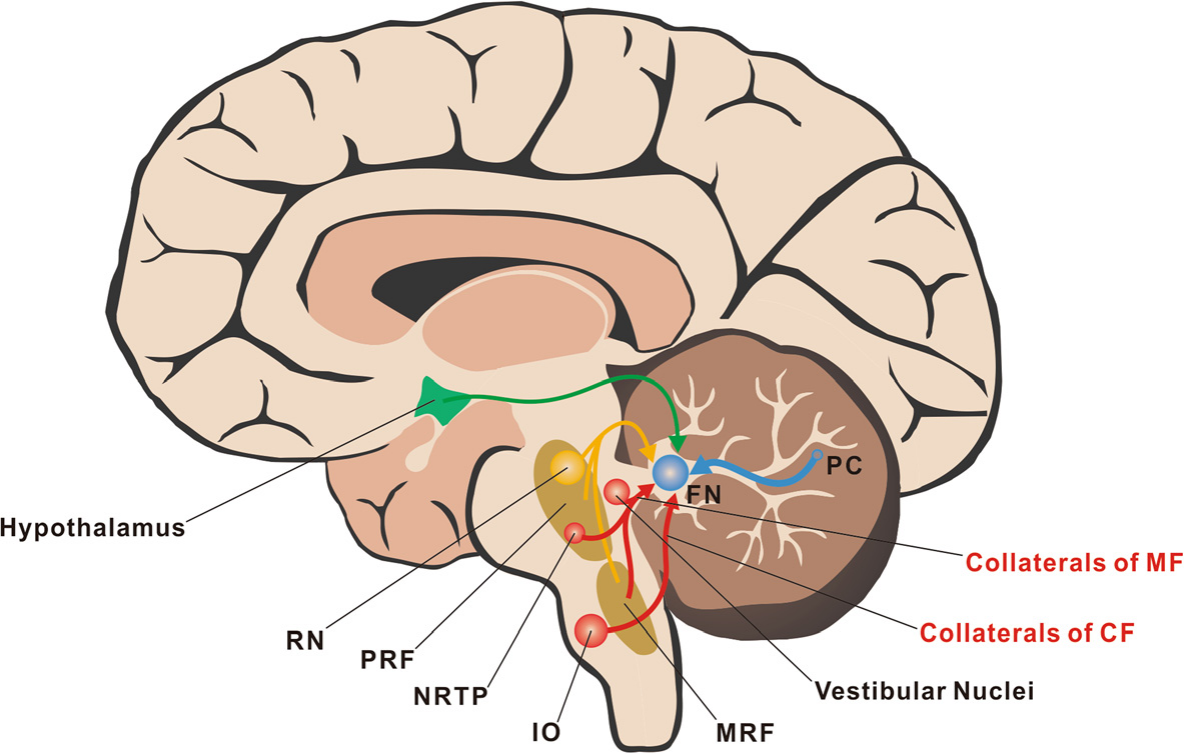
Organization of FN afferent pathways. The inhibitory GABAergic Purkinje axons constitute the most primary FN afferent pathway (*blue*). Two major types of afferents of the cerebellar circuitry, the climbing fibers from the inferior olive and the mossy fibers from the nucleus reticularis tegmenti pontis, the medullary reticular formation, and the medial vestibular nucleus send excitatory glutamatergic collaterals to FN (*red*). In addition, FN also receives serotonergic projections from the medullary/pontine reticular formation and the raphe nuclei (*yellow*) as well as histaminergic and orexinergic afferents from the hypothalamus (*green*). CF, climbing fiber; FN, fastigial nucleus; IO, inferior olive; MF, mossy fiber; MRF, medullary reticular formation; NRTP, nucleus reticularis tegmenti pontis; PC, Purkinje cell; PRF, pontine reticular formation; RN, raphe nuclei

Purkinje cells of the cerebellar vermis in both anterior and posterior lobes send inhibitory GABAergic axons to innervate FN and sculpture the FN neuronal activities [1, 20, 21]. These Purkinje cell axons transfer processed and integrated information of the cerebellar cortex of the vermis to the FN and constitute the primary FN afferent pathway (Fig. 1). Considering that the vermis receives somatic sensory inputs related to the head and proximal parts of the body from ascending spinal pathways, the FN is well known as a key component of spinocerebellum [1, 20, 22]. Interestingly, a recent anatomic study on monkeys has shown that the primary motor cortex and several cortical motor areas on the medial wall of the hemisphere provide a major source of inputs to lobules VB–VIIIB of the vermis [23], the output of which is directed largely at the FN. In addition, the FN in monkeys has also been reported to receive afferents from the flocculus, which is related chiefly to the vestibular system and mediates visual-vestibular interaction [21, 24]. Therefore, based on the afferent connections, the FN may also serve as a part of cerebrocerebellum and vestibulocerebellum.

Two major types of afferents of the cerebellar circuitry [20, 22], the climbing fibers from the caudal half of the medial and dorsal accessory subdivisions of the inferior olive [20, 22, 25] and the mossy fibers from the nucleus reticularis tegmenti pontis, the medullary reticular formation, and the medial vestibular nucleus [20, 22, 26], send excitatory glutamatergic collaterals to FN (Fig. 1). The Purkinje cell inputs combined with the afferents of collaterals of climbing/mossy fibers constitute the classical FN afferent pathways in mammals including humans. Moreover, experimental studies on opossums, cats and monkeys have revealed that FN also receives serotonergic projections arising from the medullary/pontine reticular formation and raphe nuclei [27] (Fig. 1). Noradrenergic inputs originating from the locus coeruleus to FN have been clarified in cats as well [28] (Fig. 1). Besides, it has been well demonstrated that the direct hypothalamocerebellar projections reach the FN on a variety of mammals, including primates [29]. The projections originate mainly from rostral to mid-hypothalamic levels, including the lateral hypothalamic area (LHA), the dorsal hypothalamic area, the posterior hypothalamic area, the dorsomedial hypothalamus nucleus (DMN), fascicles of the column of the fornix, and the periventricular hypothalamic nucleus (PVN) [10, 11, 29] (Fig. 1). Although the neurotransmitters used by the hypothalamocerebellar projections are still unclear, histamine and orexin are currently considered as two potential candidates [10, 11, 13, 30–32]. These monoaminergic (including serotonergic, noradrenergic and histaminergic) or neuropeptidergic afferents are often called the third type of cerebellar afferents, which may hold a key position in modulating excitability and sensitivity of FN neurons.

#### Organization of FN efferent pathways

The FN sends extensive projections to numerous motor structures via both descending and ascending pathways [22, 33]. Through the descending projections to the components of the medial descending systems in brainstem, including the vestibular nuclei and the medullary/pontine reticular formations [1, 20–22], the FN is thus considered as one of the ultimate outputs of the spinocerebellum and modulates motor behaviors via vestibulospinal and reticulospinal tracts. Moreover, the FN also targets structures in the brainstem controlling head, facial, and ocular movements, such as the cranial motor nuclei IV, VI and VII, the perihypoglossal nucleus, the rostral interstitial nucleus of the medial longitudinal fasciculus, the oculomotor and abducens nuclei, as well as the rostral interstitial nucleus of the medial longitudinal fasciculus and the paramedian pontine reticular formation in the pontine reticular formation [34]. The above descending pathways, through which FN participates in the axial, proximal and ocular motor control, have been well clarified in both primates and other mammals. Notably, in nonhuman primates, it has also been reported that via ascending pathways, axons of the FN neurons cross to the contralateral side and project to the primary motor cortex via a synapse in the ventrolateral nucleus of the thalamus [35, 36].

**Fig. 2:**
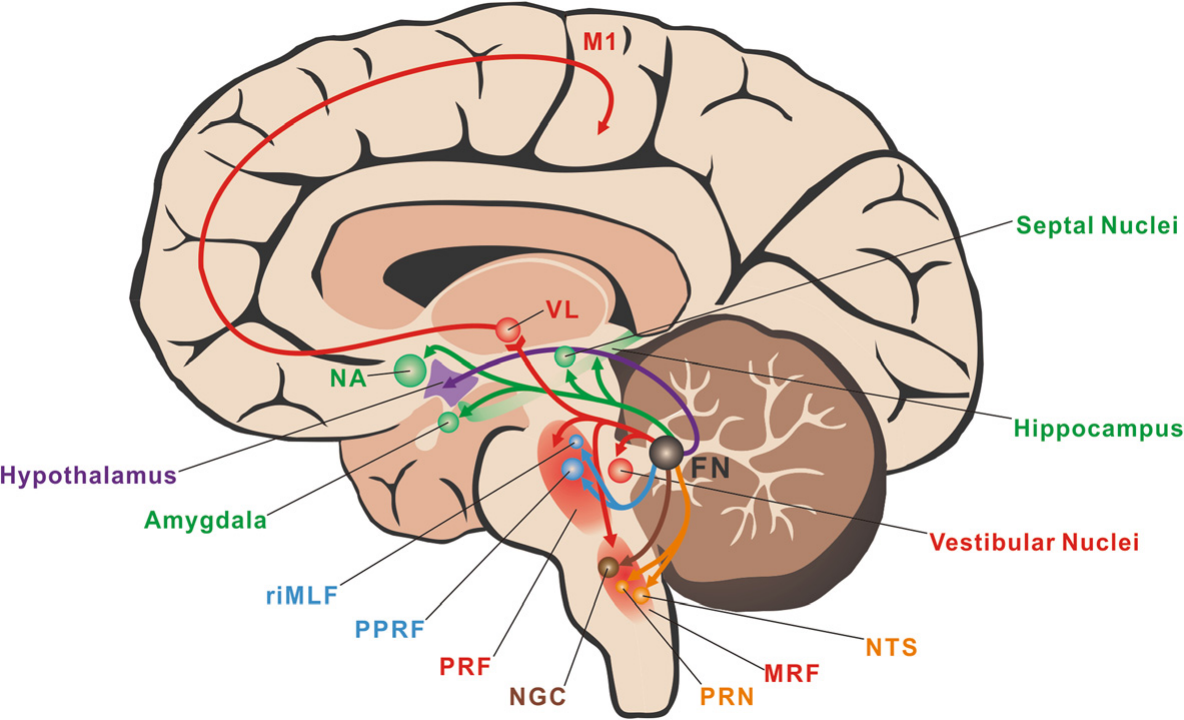
Major FN efferent pathways and their related somatic motor and nonsomatic functions. Through the descending projections to the brainstem, including the vestibular nuclei and the medullary/pontine reticular formations, and the bi-synapses ascending pathways to the primary motor cortex, FN holds a key position in axial and proximal motor control (*red*). FN also projects to the rostral interstitial nucleus of the medial longitudinal fasciculus and the paramedian pontine reticular formation in the pontine reticular formation to control ocular movement (*blue*). In addition, FN sends direct projections to the hypothalamus, visceral-related nuclei/regions in the medullary reticular formations and the limbic system to participate in feeding (*purple*), cardiovascular (*orange*), respiratory (*brown*) and emotional (*green*) regulations. FN, fastigial nucleus; M1, primary motor cortex; MRF, medullary reticular formation; NA, nucleus acumbens; NGC, gigantocellular nucleus; NTS, nucleus of solitary tract; PPRF, paramedian pontine reticular formation; PRF, pontine reticular formation; PRN, paramedian reticular nucleus; riMLF, rostral interstitial nucleus of the medial longitudinal fasciculus; VL, ventrolateral nucleus of the thalamus

In addition to motor areas, FN projects to various nonmotor structures/regions (Fig. 2). Numerous neuroanatomical studies on monkeys, beagles and cats using retrograde, anterograde tracing and autoradiography techniques have revealed that FN neurons send efferent fibers into visceral structures located within the medullary/pontine reticular formations, such as the gigantocellular nucleus (NGC), the paramedian reticular nucleus (PRN), the nucleus of solitary tract (NTS) and the nucleus ambiguus [37, 38]. The direct projections from the FN to the hypothalamus, a critical center for regulation of visceral and emotional activities, have also been well documented in a variety of mammals, including primates [10, 11, 29]. These FN neuronal axons pass through the superior cerebellar peduncle, ascendingly project to the hypothalamus, primarily reaching the LHA, DMN, ventromedial hypothalamus nucleus, medial mammillary nucleus, and PVN. Given the hypothalamic afferent inputs to the FN, there are direct bidirectional cerebellar-hypothalamic circuits bridging the FN and the hypothalamus. Besides the hypothalamus, FN also targets limbic system in monkeys and cats, including the hippocampus, dentate gyri, septal nuclei, basolateral amygdalae and nucleus acumbens, to modulate emotional activities [39, 40]. In addition, efferent pathways of the FN to the thalamus, the nigra and the ventral tegmentum of the midbrain have been reported in cats and rats [41]. The major FN efferent pathways and their related functions are summarized in Fig. 2.

### Somatic motor and nonsomatic functions of the FN

The abundant afferent and efferent connections of the FN with the extensive motor and nonmotor structures indicate that FN may be involved in various physiological functions. In fact, FN has been found to participate in not only axial, proximal and ocular motor control, but also various nonsomatic functions, including feeding, cardiovascular, respiratory, defecation and micturition, immune, and emotional regulation (Fig. 2).

#### Axial and proximal motor control

Human patients with FN lesions exhibit deficiencies principally in the control of axial and trunk muscles during attempted antigravity posture [42]. FN inactivation in primates and cats also produce severe ataxia and disturbances of equilibrium involving axial and appendicular musculature but do not influence reaching and grasping [43–45]. Moreover, a recent study indicated a disturbance of motor coordination in FN-lesioned rats, which showed poor motor performances in narrow beam-walking, grid runway and rota-rod [46]. Thus, FN holds a key position in controlling axial and proximal musculatures so as to maintain posture and control dynamic balance.

A series of electrophysiological studies has shown that neuronal discharges of FN code for proximal movement. FN neuronal discharges correlates with the force and time derivatives of trained limb movements [47]. Distinguished with the cerebellar interposed and dentate nuclei, the FN shows no detectable firing activity variations in the performance of trained wrist movements [43], which is consistent with the specialized role of FN in controlling of proximal body and limb activities rather than distal extremity movements with fine dexterity. Interestingly, it seems that only the neurons in the rostral division of FN are related to axial and proximal motor functions. Rostral division of the FN can encode the motion of the head and body in space [48–50]. By integrating the space information, rostral FN explicitly computes an internal estimate of body motion and determines spatial orientation to modulate movements related to posture and gait [48–50]. On the other hand, the bi-synaptic projections from FN to the primary motor cortex indicate that FN, one of the outputs for spinocerebellum, may also participate in movement initiation [35, 36] (Fig. 2).

Moreover, accumulating evidence has revealed that the monoaminergic afferents, including serotonergic and histaminergic, also modulate the neuronal activity of FN and consequently influence the FN-mediated motor behaviors. Both serotonin (5-HT) and histamine exert excitatory effects on FN neurons [13, 51–53]. 5-HT2A receptors contribute to the 5-HT-induced excitation on FN neurons [51]. Intriguingly, histamine selectively depolarizes projection neurons but not interneurons in the FN via the hyperpolarization-activated cyclic nucleotide-gated channels coupled to histamine H2 receptors [13]. The exclusive expression of histamine H2 receptors on glutamatergic and glycinergic projection accounts for the selective excitatory effect of histaminergic afferents in the FN. Furthermore, microinjection of 5-HT or histamine into bilateral FNs remarkably improves rat motor performances on accelerating rota-rod and balance beam, and narrows stride width in locomotion gait [52, 53]. Since serotonergic and histaminergic afferents usually form varicose contact with FN neurons and histamine H2 and 5-HT2A receptors are both metabotropic, the serotonergic and histaminergic afferent system may not transmit fast signals, but act as a biasing force to modulate their excitability and sensitivity at an appropriate level for posture and gait control [13, 31, 32].

#### Ocular motor control

Neurons of the caudal FN, known as the fastigial oculomotor region, receive information from the oculomotor vermis (lobules VI-VII) precisely predicting the real-time motion of the eye [1, 34, 54], and in turn, project to the saccade-generator circuit as well as pursuit-related structures in the lower brainstem (Fig. 2). Thus, it has long been convinced that caudal FN holds an essential role in saccadic and smooth pursuit eye movements [55–57].

Clinical evidence demonstrates that patients with midline cerebellar lesions involving the FN usually suffer from saccadic hypermetria [58]. Similarly, the saccades in experimental animals became hypermetric after caudal FN inactivation [59]. Accordingly, neurons in the caudal FN discharge in relation to saccades and encode the saccadic initiation and termination [60–62]. It has also been reported that the caudal FN neurons supply a presaccadic burst to facilitate contraversive saccades (e.g., the right caudal FN bursts before leftward saccades) and a “braking” discharge, late during the saccade, to terminate ipsiversive saccades [57, 63]. This may explain why lesions in the caudal FN cause ipsiversive saccadic hypermetria and contraversive hypometria [59, 64]. The early modulation of the caudal FN on the initiation of saccades may be mediated by the excitatory burst neurons within the brainstem reticular formation, whereas the late effect on stopping saccades may be due to the inhibitory burst neurons [57].

In the case of pursuit eye movements, caudal FN neurons discharge in an analogous way with their firing pattern during saccades, i.e., discharge early during contraversive pursuit and late for ipsilateral pursuit [65]. Consequently, a lesion in the unilateral caudal FN may result in impairment in contralateral pursuit [64–66]. However, no obvious pursuit deficits have been observed in animals or patients with bilateral caudal FN lesions [65], suggesting that caudal FN may be not necessary for intact maintenance of smooth pursuit movements.

#### Feeding control

Direct bidirectional connections between the FN and the hypothalamus [10, 11, 29] provide strong neuroanatomical substrates underlying the involvement of FN in feeding control [11, 12] (Fig. 2). Early studies have noted that FN is functional in peripheral gastrointestinal modulation. Electrical activation of the cat cerebellar FN produces modified gastric and duodenal motility [67]. Also, FN stimulation can influence on the motility in the jejunum, ileum, and colon through sympathetic and vagal pathways [68]. Furthermore, gastric motility has been clarified to be bidirectionally modulated by FN stimulation in a complex way involving adrenergic discharge, adrenal catecholamine release as well as vagal cholinergic discharge [68]. The active neurons responsible for regulation of gastrointestinal motility within the cerebellar FN are restricted to the rostral ventromedial region [68].

More recent studies have revealed that the cerebellar FN can regulate hypothalamic feeding-related neurons through direct cerebellohypothalamic projections [11, 12]. Stimulation of the FN in rats or cats was found to evoke postsynaptic responses and to modulate the activity of LHA glucose-sensitive/glycemia-sensitive neurons, which may sense the blood glucose level and subsequently trigger multiple visceral-somatic responses (e.g., initiating or ceasing food intake) [69–71]. There also exist functional connections between the FN and the glycemia-sensitive neurons in the DMN, another hypothalamic region implicated in adiposity signaling and feeding regulation [72]. Moreover, besides blood glucose signal, the cerebellar FN inputs also converge and integrate with other important peripheral feeding-associated signals including the gastric vagal and leptin inputs on single hypothalamic DMN neurons [72]. Considering that gastric vagal inputs, leptin and blood glucose level are feeding-related visceral signals and FN may forward the somatic information to the hypothalamus, it can be speculated that an integration of the somatic-visceral response related to the food intake may take place in the cerebellar FN-hypothalamic circuits and play an important role in the short-term or even long-term regulation of feeding behavior.

#### Cardiovascular control

FN sends projections to the NTS and the PRN of the medulla, both of which mediate the baroreceptor reflex [38], suggesting a direct involvement of FN neurons in cardiovascular functions (Fig. 2). In fact, fastigial pressor response (FPR) has been found early in the 1960s, as fastigial stimulation was noticed to cause a rapid rise of arterial pressure in cats [73]. Thereafter, cardiovascular responses, i.e., the elevations in arterial pressure, heart rate and regional cerebral blood flow elicited by electrical stimulation of the FN, were found in various species, including monkeys, dogs, rabbits, ferrets and rats [74]. In recent years, MRI studies visualized signal changes of FN after pharmacological pressor and depressor challenges in developing and adult animals, also supporting a functional role of FN in cardiovascular control [75].

The FPR is abolished by bilateral lesions of either the PRN [73, 76] or the rostral ventrolateral reticular nucleus of the medulla oblongata (RVLM) [77]. Since direct FN afferent projections have been identified exclusively in the PRN rather than RVLM, the FPR is speculated to be a consequence of monosynaptic activation of sympathetic vasomotor neurons in the PRN and polysynaptic excitation of reticulospinal sympathoexcitatory axons of RVLM. In addition, besides a typical rise in blood pressure, renal sympathetic nerve activity can be concomitantly increased after stimulation of the FN in anesthetized animals [78].

Intriguingly, animals with FN lesions have no significant disorder in resting blood pressure or heart rate, but exhibit remarkable defects in compensatory responses to hemorrhage or endotoxic shock to the point that a fatal outcome can ensue [79]. Moreover, deactivation and activation of FN neuronal activity was observed to occur immediately after the elevation and lowering of arterial pressure by phenylephrine and nitroprusside, respectively [80]. All these findings indicate that FN may serve a sympathoexcitatory role for hypotensive challenges and sympathoinhibitory capacity for pressor challenges, and thereby may play an essential role in the compensation of cardiovascular activities to large blood pressure changes rather than the maintenance of vascular tone at rest.

#### Respiratory control

Electrical or chemical stimulation of the cerebellar FN in anesthetized animals have been reported to elicit significant respiratory responses, including an elevation of respiratory frequency and inspiratory flow as well as an earlier-onset of phrenic nerve discharge [81]. In humans, electrical stimulation within the vicinity of the FN during surgery results in respiratory tachypnea. [82] Moreover, a c-Fos immunostaining study in animals [83] and a MRI study in humans [84] revealed that the neuronal activity of FN remarkably increased in response to hypercapnia. Lesion studies showed that ablation of the FN did not significantly alter eupneic breathing, but did markedly attenuate the respiratory response to medium and severe hypercapnia as well as hypoxia [81]. Therefore, similar to the FPR, which is contribute to the compensation of large cardiovascular alternation rather than maintenance of resting blood pressure, the FN-mediated respiratory response may be more critical for the facilitation of stressed breathing, but not for the eupneic ventilation maintenance.

Interestingly, although FN is involved in the modulation of stressed breathing, it exhibits no functional regulation on respiratory mechanoreflexes. Ablation of FN does not significantly alter respiratory responses elicited by manipulation of bronchial and pulmonary mechanoreceptors by applying lung inflation [85], inspiratory occlusion/airway resistance [86], and/or probing the intrathoracic trachea. [87] In contrast, FN holds an important position in regulation of respiratory chemoreflexes. FN lesions attenuate the respiratory responses to hypercapnia and hypoxia [88]. Elevation of CO_2_/H^+^ within the FN facilitates respiratory output in anesthetized rats [89]. In awake animals, focal acidosis in the rostral FN also significantly increases inspired ventilation, whereas focal acidosis in the caudal FN significantly decreases inspired ventilation [90], indicating that there is a heterogeneous population of CO_2_/H^+^ chemoreceptor neurons in the FN that affect respiratory control. Furthermore, lesioning the FN attenuates CO_2_/H^+^ ventilatory sensitivity during wakefulness [91]. All these studies imply a CO_2_/H^+^ chemoreception existed in FN neurons and an active modulation of the cerebellar nucleus on respiratory responses.

Anatomically, FN neurons send efferent fibers to a variety of pontomedullary nuclei recognized to be involved in respiratory modulation, such as the ambiguous nucleus, pontine respiratory groups, NGC, and PRN [38]. Thus, the possible respiratory pathways underlying the FN-mediated respiratory responses were examined by separate ablation of these FN targeted respiratory centers. It has been shown that destruction of neurons within the pontine respiratory groups, Bötzinger complex, and PRN fails to alter respiratory responses induced by FN stimulation [92]. In contrast, respiratory modulation of FN totally disappeared after selective ablation of bilateral NGC neurons [89], suggesting that FN-mediated respiratory response depends on the relay of NGC (Fig. 2).

#### Other visceral, immune, and emotional functions

The FN has also been implicated in defecation and micturition regulations [93]. FN stimulation regularly suppresses both somatomotor and autonomic components of the defecation reflex, but influences the bladder motility in a bidirectional manner, either suppressed or enhanced micturition reflex, depending both on prevailing bladder tone and on the fastigial site stimulated [93]. Moreover, an involvement of FN in immune regulation via the cerebellum-hypothalamus-sympathetic nerves-lymphocytes pathway has been noticed in FN lesion rats in which both T lymphocyte proliferation and the natural killer cell cytotoxicity are dramatically enhanced [94]. Intriguingly, stimulation of the FN in cats determines fits of anger [95], indicating FN may be also involved in emotional regulation. Since there are direct anatomic linkages between the FN and the limbic system, including the hypothalamus, septal nuclei, hippocampus, nucleus acumbens, and amygdala [10, 11, 29, 39, 40], the FN, together with the vermis and flocculonodular lobe, is considered as the limbic cerebellum which serves as an extension of Papez circuit [96, 97] (Fig. 2).

### The clinical implication of FN in SCAs and nonmotor diseases

Since FN is actively involved in the regulation of various important physiological functions, its lesion or dysfunction leads to many motor and nonmotor symptoms and syndromes. SCAs are a well-known clinically heterogeneous group of disorders characterized by cerebellar syndrome, including ataxia of gait (usually the main ataxic sign), posture ataxia, dysmetria and/or kinetic tremor in four limbs, as well as oculomotor deficits (nystagmus, hypermetria/hypometria of saccades) [98]. In recent years, clinical studies have explored the topography of ataxia symptoms in human cerebellum [55, 99–101]. Studies on subjects with chronic focal lesions after cerebellar tumor resection have demonstrated that ataxia of gait and posture is associated with lesions affecting the FN [42, 102]. In a gait ataxia study, lesion-based MRI subtraction analysis has shown that the FNs are more frequently affected in patients with impaired compared to unimpaired dynamic balance in gait [42]. And in a study on posture ataxia, MRI overlays have also revealed that the FNs are damaged in all the tested cerebellar tumor resection patients manifested abnormal postural sway [102]. Moreover, the correlation with clinical ataxia rating scores and MRI-defined lesions has been performed in patients with acute and chronic ischemic/surgical cerebellar lesions using voxel-based lesion-symptom mapping, and ataxia of posture and gait has been found to be highly correlated with lesions of the FN [103]. On the other hand, the FN-related saccadic deficits occur in numerous families of SCAs. Degeneration in the fastigial oculomotor region causes the saccadic dysmetria in both the patients with SCA type 6 (SCA6) and those with a similar phenotype but without the SCA6 genetic mutation (so-called late onset cerebellar ataxia; LOCA) [104]. In addition, FN has been proposed as a central target for memantine, a non-competitive blocker of the NMDA receptor, in the treatment of patients of SCA with saccadic intrusions (SCASI) to relieve their symptoms of saccadic intrusions [105].

Besides SCAs, FN has also been reported to be related with the pathology of various nonmotor diseases. Congenital central hypoventilation syndrome (CCHS) is a life-threatening disorder involving an impaired ventilatory response to hypercarbia and hypoxemia. MRI studies have demonstrated that FN in CCHS patients respond inappropriately to ventilatory or blood pressure challenges [106–108]. Moreover, neural damage in FN has been observed in CCHS subjects [109]. These findings are consistent with the evidence from animal experiments indicating that the FN plays a major role in modulating breathing patterns, especially respiratory chemoreflexes. Furthermore, some recent studies in rats have shown that electrical or chemical stimulation of FN plays protective effect on stress gastric mucosal injury and acute myocardial infarction [110, 111], indicating a possible involvement of FN in the pathogenesis and treatment of gastrointestinal disorders. Interestingly, abnormalities in the FN have also been observed among patients with autism and cerebellar cognitive affective syndrome [112, 113], strongly suggesting that FN dysfunction may be closely related to syndromes of affective disturbance.

## Conclusion

As the most conservative nucleus in the cerebellum, the FN holds a key position in the spinocerebellar circuits and functions. It is not a simple relay station in the spinocerebellum but an important node in the cerebellar circuitry integrating the GABAergic Purkinje inputs from the cortex of vermis, the glutamatergic inputs from collaterals of mossy and climbing fibers, and the monoaminergic/neuropeptidergic signals from the third type of cerebellar afferents. The FN glutamatergic, glycinergic and GABAergic projection neurons send the final integrated information out of the spinocerebellum. Via projecting to the medial descending systems of the brainstem and eye movement related central structures, the FN contributes to the axial, proximal and ocular motor control. Furthermore, by directly connecting extensive nonmotor structures, particularly the hypothalamus, the FN also actively participates in various nonsomatic functions, such as feeding control, cardiovascular and respiratory regulation, defecation and micturition, immune, as well as emotions. Intriguingly, stimulation of the FN in conscious animals elicits an integrated response, i.e., hypertension and tachypnea accompanied by complex behaviors, such as grooming, biting, and eating [114]. Also, the FN is particularly critical for regulating cardiovascular and respiratory functions in exercising animals [115, 116]. Dysfunction or lesion of FN may result in not only ataxias and oculomotor deficits, but also nonmotor symptoms involving cardiovascular, respiratory and emotional disorders. Therefore, the FN may function as an essential central component involved in somatic-nonsomatic integration, which is critical for generation of a coordinated behavioral response for adapting to changes of internal and external environments.
